# Monthly Summary

**Published:** 1872-10

**Authors:** 


### MONTHLY SUMMARY.

*Cancer from a Blow.*—Two negroes became pugilistic; one struck the other a heavy blow with a weight, on the upper lip, directly under his nose, which of necessity came in contact with his teeth. The lip was mashed into a black jelley; his front teeth were caved in. He replaced his teeth, and was able to masticate with them in a short time, although they remained loose to some extent. This was three years ago. The teeth gave him occasional pain, and in time there was a fungus growth on the gums. I saw the patient first about September of last year; examined the parts, and was not entirely satisfied as to the nature of the difficulty. I did nothing at the time; was informed by the patient that some portions of the fungus had been removed by another physician. I explained to him about the nature of the difficulty, and what the operation would be. He left, and I saw him no more for two months. When next I saw him I felt confident that it was a cancer, involving the injured parts, and also the superior maxillary and bones of the nose. The mouth was rapidly filling up with fungus He consented to an operation. Drs. M. S. Thomas and Van Duyn assisted. I removed six front teeth, and then made a section of the superior maxillary. Here we rested.

After carefully surveying the ground we concluded not to interfere further, as another operation would necessitate the removal of the whole of the superior maxillary, nasal bones, and in all probability, his eyes—then with no prospect of a permanent cure. We used no chloroform. In a very short time the mouth began to fill again with the nodular tissue. a part of which I again removed. It is very surprising how very rapidly it grows. I consider this case one of simple injury, of a local character, one that would have insured success by an early operation What gave rise to cancer in this case, I am sure I can't tell you. I know there was a constant source of irritation; but why not necrosis or caries, instead of cancer?—*Dr. Brock, in Med. Herald.*

---

*Alcohol as a Medicine.*—Dr. Forbes Winslow, in London *Times*, states that during the last twenty years he has seen numerous cases, more particularly among women, of an insane craving for alcohol, which could be traced to the injudicious use of stimulants given, in the first instance, medicinally. The unwise and prolonged continuance of the use of alcohol after the physician has retired from the treatment of the case, Dr. Forbes Winslow says, causes spirits to become a necessity of life, inducing habits of tippling and confirmed drunkenness, and eventually developing severe diseases of the brain and mind, and frightful disorders of the nervous system. Dr. Forbes Winslow speaks in the strongest terms of the condemnation of the stimulating theory as applied to the treatment of acute inflammatory affections. He says that the habit of stimulation may be established by the occasional sipping of spirits of wine, cologne water, spirits of chloroform, spirits of ammonia or any of the medicinal tinctures, as well as by drinking brandy, whisky or wine. Such opinions are not yet indorsed by the profession at large with us.—*Medical Examiner.*

---

*Color Blindness.*—At a recent meeting of the Boston Society of Natural History, the methods of testing patients for color blindness, and for loss of the power of color perception, were explained. An instrument, invented in Germany, for testing color blindness, was exhibited and explained. This instrument consists of a rotating apparatus, which moves a disk whose center is a circle, one-half black and the other white; outside of this is a ring half red and half green, then another ring of violet and red, then the outside ring of violet and green. When rapidly rotated the center appears to be colored gray; that is, black and white mixed. To a *green* blind person the middle ring will appear gray, that being the result to him of a mixture of violet and red. The outer ring will appear gray to the *red* blind patient, and the inner gray to the *violet* blind. By the use of this instrument it is evident a large number of patients may be simultaneously examined for one or more kinds of color blindness.—*Medical and Surgical Reporter.*

---

*Transplantation of Mucous Membrane.*—At a meeting of the College of Physicians and Surgeons of Vienna, Dr. Czerny, *Am. Practitioner*, exhibited a case of Transplantation of mucous membrane of the nose upon a granulating surface of the upper arm. He spoke of three cases he had experimented on in this way, and one in which he had taken the mucous membrane from the mouth. The mucous membrane in one instance was taken from an extirpated polypus, and transplanted two hours after its removal. It had lost on its new ground its villi, and had changed into basement epithelium.—*N. Y. Med. Jour.*

